# Early arterial blood gas parameters, clinical features, and neuroimaging findings in neonates undergoing therapeutic hypothermia for suspected hypoxic-ischemic encephalopathy: a retrospective single-center study

**DOI:** 10.3389/fped.2026.1826725

**Published:** 2026-07-09

**Authors:** Ece Gültekin

**Affiliations:** Department of Pediatric Neurology, Biruni University Faculty of Medicine, İstanbul, Türkiye

**Keywords:** blood gas analysis, hypoxia-ischemia, brain, infant, newborn, magnetic resonance imaging, therapeutic hypothermia

## Abstract

**Background:**

Hypoxic-ischemic encephalopathy (HIE) remains an important cause of neonatal mortality and long-term neurodevelopmental impairment. Although therapeutic hypothermia is the standard treatment for term and near-term infants with suspected moderate-to-severe HIE, the relationships among early blood gas parameters, clinical findings, and radiologic abnormalities in cooled neonates are not fully defined.

**Methods:**

This retrospective observational study included neonates aged 0–28 days who underwent therapeutic hypothermia for suspected HIE at any neonatal intensive care unit and were subsequently evaluated by the Department of Pediatric Neurology at Biruni University Hospital. Demographic and perinatal characteristics, blood gas parameters, seizure status, electroencephalographic findings, antiepileptic treatment, cranial ultrasonography, and cranial MRI findings were obtained from medical records. Final HIE classification was assigned retrospectively after review of the clinical course, documented seizures, serial neurologic findings, electroencephalographic data, and neuroimaging findings.

**Results:**

A total of 22 neonates were included. Final HIE classification identified no final evidence of HIE in 16 patients (72.7%), stage 1 HIE in 1 (4.5%), stage 2 HIE in 3 (13.6%), and stage 3 HIE in 2 (9.1%). Compared with neonates without HIE, those with any HIE had significantly lower bicarbonate levels (*p* = 0.010). Blood gas pH and 5 min Apgar scores also tended to be lower in the HIE group, although without statistical significance. Neonates with seizures had lower gestational age (*p* = 0.002) and more negative base excess values (*p* = 0.029). Concordance between cranial ultrasonography and MRI was low; many neonates with normal ultrasonography had abnormal MRI findings.

**Conclusions:**

Lower bicarbonate levels were associated with the presence of any-stage HIE compared with no final evidence of HIE, while more severe metabolic acidosis was associated with seizure occurrence. The bicarbonate finding was consistent with previously established evidence and should be interpreted as supportive rather than novel. The discordance between cranial ultrasonography and MRI was consistent with the previously established greater sensitivity of MRI and was considered a supportive secondary finding.

## Introduction

Hypoxic-ischemic encephalopathy (HIE) remains a major cause of neonatal mortality and long-term neurodevelopmental impairment worldwide. Early identification of infants at risk is crucial, as timely recognition may facilitate appropriate monitoring, prognostic assessment, and initiation of neuroprotective interventions. Among the earliest available objective markers, umbilical cord and early postnatal blood gas parameters provide important information on perinatal acid-base status and the severity of hypoxic-ischemic insult. Previous studies have shown that severe acidosis, reflected by low arterial pH and marked base deficit, is associated with more severe neurologic injury and adverse neonatal outcomes. In addition, blood gas abnormalities obtained during the early postnatal period have been correlated with the severity of brain injury detected on magnetic resonance imaging (MRI) in infants evaluated for therapeutic hypothermia (1–3).

Therapeutic hypothermia is currently the standard neuroprotective treatment for term and near-term neonates with moderate to severe HIE when initiated within the recommended therapeutic window. Landmark clinical trials and MRI-based follow-up studies have shown that hypothermia reduces the extent of brain injury and improves survival without moderate-to-severe disability in appropriately selected infants. Brain MRI has therefore become a key tool not only for confirming the pattern and severity of hypoxic-ischemic injury, but also for estimating prognosis after neonatal encephalopathy. Importantly, abnormal MRI patterns remain strongly associated with adverse neurodevelopmental outcomes even in infants treated with hypothermia (4–6).

Despite these advances, the relationship between early biochemical markers, bedside clinical findings, and radiologic abnormalities is not always straightforward in routine neonatal intensive care practice. Seizures, EEG abnormalities, and cranial ultrasonography findings may not fully reflect the extent of injury identified on MRI, and the degree to which early blood gas abnormalities correspond to imaging findings may vary across patient populations. Recent retrospective studies have continued to show associations between early blood gas derangement, MRI-defined injury severity, and short-term neurologic outcomes in neonates assessed for or treated with hypothermia (1, 3, 7).

In this context, retrospective evaluation of infants managed with therapeutic hypothermia may provide clinically useful data regarding the correlation of blood gas parameters with HIE stage, neurologic manifestations, and radiologic findings. Such analyses may help refine early risk stratification and improve decision-making in neonatal intensive care units. Therefore, the present study aimed to investigate the associations among umbilical cord arterial or early postnatal arterial blood gas parameters, clinical findings, and cranial imaging results in neonates who underwent therapeutic hypothermia for suspected or confirmed HIE at different neonatal intensive care units and were subsequently evaluated by the Department of Pediatric Neurology at Biruni University Hospital.

## Materials and methods

### Study design and setting

This retrospective observational study was conducted in the Department of Pediatric Neurology at Biruni University Hospital. The study was approved by the Biruni University Scientific Research Ethics Committee (Decision No: 2024-BIAEK/14-13; Date: 20 October 2025). The study was conducted in accordance with the ethical principles of the Declaration of Helsinki and the Good Clinical Practice guidelines. Written informed consent for diagnostic and therapeutic procedures, as well as for the use of clinical data for scientific purposes, had been obtained from the families of the patients in accordance with institutional practice. Because of the retrospective design of the study, all data were collected from existing medical records, anonymized before analysis, and handled in accordance with confidentiality and data protection principles.

The study included neonates aged between 0 and 28 days who were evaluated for suspected HIE and underwent therapeutic hypothermia during the study period. Medical records, laboratory findings, neurologic assessments, and radiologic imaging reports of all eligible patients were reviewed retrospectively.

### Study population

**Figure 1:**
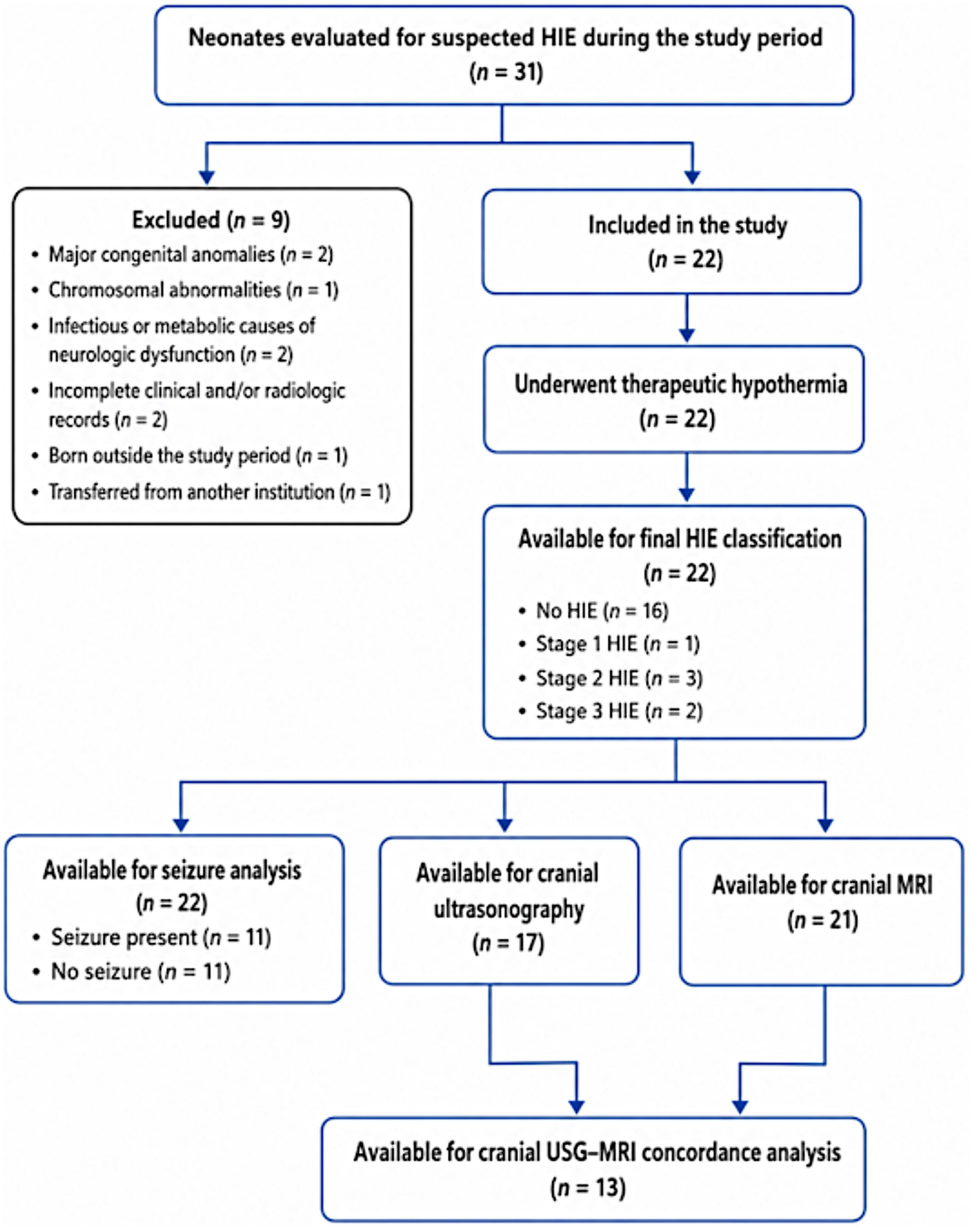
Flow diagram of patient selection and data availability for the study analyses.

Neonates were included in the study if they met all of the following criteria: gestational age of at least 36 weeks, postnatal age of 6 h or less at the initiation of therapeutic hypothermia, treatment with therapeutic hypothermia for suspected hypoxic-ischemic encephalopathy, and availability of blood gas, clinical, and radiologic data. Patients with major congenital anomalies, chromosomal abnormalities, infectious or metabolic causes of neurologic dysfunction, incomplete clinical and/or radiologic records, and those born outside the study period or transferred from another institution were excluded. The patient selection process, reasons for exclusion, and availability of data for the principal analyses are summarized in Figure 1.

Eligibility for therapeutic hypothermia was assessed according to the institutional neonatal intensive care protocol. Therapeutic hypothermia was initiated in neonates with evidence of an acute perinatal hypoxic-ischemic event and at least one of the following findings: an Apgar score of 5 or lower at 10 min, continued need for resuscitation or assisted ventilation at 10 min, an umbilical cord or early postnatal blood gas pH of 7.00 or lower, or a base deficit of 16 mmol/L or greater. In addition, eligible neonates were required to demonstrate clinical findings compatible with moderate-to-severe neonatal encephalopathy, including abnormalities in consciousness, spontaneous activity, posture, muscle tone, primitive reflexes, autonomic function, and/or seizures. The final decision to initiate therapeutic hypothermia was made by the attending neonatologist based on the combined assessment of perinatal, biochemical, and neurologic findings.

### Initial eligibility assessment and final HIE classification

Eligibility for therapeutic hypothermia and final HIE classification were considered separate clinical constructs. Therapeutic hypothermia was initiated within 6 h after birth when the attending neonatologist identified an acute perinatal hypoxic–ischemic event together with predefined biochemical or resuscitation criteria and neurologic findings suggestive of moderate-to-severe neonatal encephalopathy.

The severity of neonatal encephalopathy during the acute clinical course was assessed using the modified Sarnat framework, which evaluates level of consciousness, spontaneous activity, posture, muscle tone, primitive reflexes, autonomic function, and seizure occurrence. The neurologic examination documented before cooling or during the first 6 postnatal hours formed the basis of the acute encephalopathy assessment.

Because the present study was retrospective, a final diagnostic classification was subsequently assigned after review of the complete hospital record, including the early neurologic examination, serial neurologic findings, documented clinical or electrographic seizures, electroencephalographic findings, neuroimaging results, and the overall clinical course. Neonates were categorized as having no final evidence of HIE or stage 1, stage 2, or stage 3 HIE. This final classification represented an integrated retrospective diagnostic assessment and was distinct from the acute modified Sarnat assessment performed before or during initiation of therapeutic hypothermia. The category “no final evidence of HIE” did not represent a healthy control group; it included neonates who fulfilled the institutional criteria for initiation of therapeutic hypothermia because of suspected HIE but whose subsequent clinical, electroencephalographic, and neuroimaging evaluation did not support a final diagnosis of HIE. Accordingly, comparisons between neonates with no final evidence of HIE and those with any-stage HIE were considered exploratory within a treatment-selected cohort. The relatively high proportion of neonates classified as having no final evidence of HIE reflects the fact that therapeutic hypothermia was initiated prospectively during the narrow treatment window on the basis of suspected injury, whereas the final classification was assigned retrospectively after the complete clinical, electroencephalographic, and neuroimaging course had become available.

### Data collection

Data were obtained from hospital medical records and archived imaging reports. The recorded variables included sex, mode of delivery, gestational age, birth weight, birth length, head circumference, Apgar score at 1 min, Apgar score at 5 min, HIE stage, blood gas parameters, seizure status, electroencephalographic findings, antiepileptic treatment, cranial ultrasonography findings, cranial MRI findings, and available follow-up data.

For blood gas analysis, the earliest available umbilical cord arterial or early postnatal arterial blood gas sample obtained within the first hour after birth and before the initiation of therapeutic hypothermia was used. When both sample types were available, the umbilical cord arterial sample was prioritized. Of the 22 samples included in the analysis, 15 were umbilical cord arterial samples and 7 were early postnatal arterial samples. No venous or capillary blood gas samples were included. The recorded blood gas parameters were pH, partial pressure of carbon dioxide, bicarbonate concentration, and base excess.

Neonatal seizure occurrence was determined from contemporaneous medical records. A seizure was considered present when a clinical event documented by the attending neonatologist or pediatric neurologist was considered compatible with a neonatal seizure and/or when an electrographic seizure was identified on conventional electroencephalography or amplitude-integrated electroencephalography. Events described only as nonspecific movements, jitteriness, or tremor without a clinical diagnosis of seizure were not classified as seizures. Because continuous electroencephalographic monitoring was not uniformly available, the seizure variable represented documented clinical and/or electrographic seizures rather than exclusively electrographically confirmed seizures. The need for antiepileptic treatment was also recorded from the medical records.

Radiologic evaluation consisted of cranial ultrasonography and cranial MRI performed during hospitalization. Cranial MRI was performed after completion of the 72-hour therapeutic hypothermia period and controlled rewarming, generally between postnatal days 4 and 7. When more than one cranial MRI examination was available, the first MRI performed after rewarming was included in the analysis. Cranial ultrasonography was performed during hospitalization according to the clinical condition of the neonate. All radiologic findings were reviewed according to the official radiology reports recorded in the hospital information system.

All included neonates underwent therapeutic hypothermia according to the routine clinical practice of the unit. The target rectal temperature, duration of cooling, and associated clinical findings were obtained from patient records.

### Outcome measures

The primary objective of the study was to investigate the relationship between blood gas parameters and final HIE classification. Secondary objectives were to describe seizure occurrence, electroencephalographic abnormalities, cranial ultrasonography findings, cranial MRI findings, and antiepileptic treatment according to final HIE classification. In addition, concordance between cranial ultrasonography and cranial MRI findings was assessed in patients with both imaging modalities available.

### Statistical analysis

Statistical analyses were performed using IBM Statistical Package for the Social Sciences for Mac, version 26.0 (IBM Corp., Armonk, New York, United States of America), and R software, version 4.3.2 (R Foundation for Statistical Computing, Vienna, Austria). Distribution of continuous variables was assessed using visual methods and analytical tests, including histogram evaluation and the Shapiro–Wilk test. Continuous variables were expressed as mean and standard deviation for normally distributed data and as median and interquartile range for non-normally distributed data. Categorical variables were expressed as number and percentage.

Comparisons between two independent groups were performed using the Mann–Whitney *U* test for continuous variables and the chi-square test or Fisher's exact test for categorical variables, as appropriate. Agreement between cranial ultrasonography and cranial MRI findings was assessed descriptively and by Cohen's kappa coefficient when applicable. Exploratory univariable Firth penalized logistic regression analyses were performed in R using the “logistf” package to evaluate factors associated with any-stage HIE and neonatal seizure occurrence. For any-stage HIE, bicarbonate level, blood gas pH, base excess, 5 min Apgar score, and gestational age were assessed as candidate predictors. For neonatal seizures, gestational age, base excess, 5 min Apgar score, pCO_2_, bicarbonate level, and the presence of any-stage HIE were evaluated. Each predictor was entered into a separate univariable model, and the results were reported as odds ratios with 95% confidence intervals. Multivariable regression analysis was not performed because of the limited sample size and small number of outcome events, which would have increased the risk of overfitting and unstable estimates. A two-sided *p* value of less than 0.05 was considered statistically significant.

The sample size calculation was based on the primary comparison of bicarbonate levels between neonates with no HIE and those with any-stage HIE. Assuming a clinically relevant between-group difference of 4.0 mmol/L, a common standard deviation of 3.3 mmol/L, a two-sided alpha level of 0.05, 80% statistical power, and an expected allocation ratio of 3:1, a minimum total sample size of 20 neonates was required, including 15 neonates with no HIE and 5 neonates with any-stage HIE.

## Results

The study population comprised 22 neonates. Males were slightly more frequent than females (54.5% vs. 45.5%). Cesarean delivery was performed in 54.5% of cases. Almost all infants were born at term (95.5%). The majority had a birth weight of 2,500–3,999 g (72.7%), while 18.2% had a birth weight ≥4,000 g. Birth length was ≥50 cm in 54.5% and head circumference was ≥35 cm in 59.1% of neonates. Apgar scores indicated notable perinatal compromise, with 54.5% of infants having a 1 min Apgar score ≤3; however, by 5 min, most infants had improved to an Apgar score of 4–6 (54.5%), and 27.3% had a score ≥7 (see Table 1).

**Table 1:** Demographic, perinatal, and birth characteristics of the study population.

Variable	Category	*n* (%)
Sex	Male	12 (54.5)
	Female	10 (45.5)
Mode of delivery	Cesarean section	12 (54.5)
	Normal spontaneous delivery	10 (45.5)
Gestational age	Preterm (<37 weeks)	1 (4.5)
	Term (≥37 weeks)	21 (95.5)
Birth weight	<2,500 g	2 (9.1)
	2,500–3,999 g	16 (72.7)
	≥4,000 g	4 (18.2)
Birth length	<50 cm	10 (45.5)
	≥50 cm	12 (54.5)
Birth head circumference	<35 cm	9 (40.9)
	≥35 cm	13 (59.1)
Apgar score at 1 min	≤3	12 (54.5)
	4–6	8 (36.4)
	≥7	2 (9.1)
Apgar score at 5 min	≤3	4 (18.2)
	4–6	12 (54.5)
	≥7	6 (27.3)

Of the 22 neonates, 16 (72.7%) had no HIE, while 1 (4.5%), 3 (13.6%), and 2 (9.1%) were classified as HIE stage 1, 2, and 3, respectively. All 16 neonates in the no-final-evidence-of-HIE group had initially fulfilled the institutional criteria for therapeutic hypothermia because of suspected hypoxic-ischemic injury; however, the subsequent integrated clinical, electroencephalographic, and neuroimaging assessment did not support a final diagnosis of HIE. Blood gas analysis revealed severe acidosis in a considerable proportion of patients: pH was 6.80–6.99 in 63.6% and <6.80 in 4.5%, whereas 68.2% had a pCO_2_ level ≥60 mmHg. HCO_3_ was <10 mmol/L in 50.0% of cases, and base excess was ≤−20 in 27.3%, supporting the presence of substantial metabolic derangement. The target rectal temperature was 33.5 °C in all neonates (see Table 2).

**Table 2:** HIE stage, blood gas parameters, and target rectal temperature of the study population.

Variable	Category	*n* (%)
HIE stage	None	16 (72.7)
	Stage 1	1 (4.5)
	Stage 2	3 (13.6)
	Stage 3	2 (9.1)
Blood gas pH	<6.80	1 (4.5)
	6.80–6.99	14 (63.6)
	≥7.00	7 (31.8)
pCO_2_	<35 mmHg	2 (9.1)
	35–59.9 mmHg	5 (22.7)
	≥60 mmHg	15 (68.2)
HCO_3_	<10 mmol/L	11 (50.0)
	10–15 mmol/L	7 (31.8)
	>15 mmol/L	4 (18.2)
Base excess (BE)	≤−20	6 (27.3)
	−19.9 to −10	11 (50.0)
	>−10	5 (22.7)
Target rectal temperature	33.5 °C	22 (100.0)

Neuroimaging and neurologic findings varied according to HIE stage. In the no-HIE group (*n* = 16), cranial ultrasonography findings were predominantly normal, whereas cranial MRI findings were heterogeneous and included hemorrhagic, ischemic, white matter, basal ganglia, and posterior fossa abnormalities. Seizures were observed in several patients in this group, while EEG findings were mostly normal or unavailable, with occasional epileptiform or seizure activity. Antiepileptic treatment in the no-HIE group was variable and ranged from no treatment to phenobarbital, levetiracetam, or combined phenobarbital plus levetiracetam therapy. In the single patient with stage 1 HIE, no abnormal neuroimaging, seizure, EEG, or treatment findings were noted. Among patients with stage 2 HIE (*n* = 3), cranial ultrasonography and MRI were mostly normal, although one patient had posterior fossa CSF space prominence and cavum septum pellucidum. Seizures and bilateral focal sharp waves on EEG were each present in one patient. In stage 3 HIE (*n* = 2), severe neuroimaging abnormalities were observed in both patients, and seizures were present in all cases; antiepileptic treatment included levetiracetam alone or combined phenobarbital plus levetiracetam (see Table 3).

**Table 3:** Neuroimaging, seizure, EEG, and antiepileptic treatment characteristics according to HIE stage.

HIE stage	Cranial USG findings	Cranial MRI findings	Seizures	EEG findings	Antiepileptic treatment
No HIE (*n* = 16)	Predominantly normal; isolated nonspecific abnormalities or missing data were noted in a few patients.	Heterogeneous findings, ranging from normal MRI to hemorrhagic, ischemic, white matter, basal ganglia, and posterior fossa abnormalities.	Variable; present in several patients and absent in others.	Mostly normal or unavailable; occasional epileptiform activity or seizure activity was observed.	Variable; no treatment, PB, LEV, or PB + LEV.
Stage 1 (*n* = 1)	No abnormality detected.	No abnormality detected.	Absent.	No abnormality detected.	None.
Stage 2 (*n* = 3)	Mostly normal; unavailable in 1 patient.	Mostly normal; posterior fossa CSF space prominence and cavum septum pellucidum were noted in 1 patient.	Present in 1 patient.	Bilateral focal sharp waves were observed in 1 patient.	PB in 1 patient, PB + LEV in 1 patient, and no antiepileptic treatment in 1 patient.
Stage 3 (*n* = 2)	Abnormal or unavailable; increased parenchymal echogenicity and intraventricular hemorrhage were noted in 1 patient.	Marked abnormalities were present in both patients, including bilateral multicystic changes with hemorrhagic signal abnormalities and bilateral parieto-occipital subdural hematoma.	Present in both patients.	No abnormality reported.	LEV in 1 patient and PB + LEV in 1 patient.*

* One patient also received midazolam infusion, phenytoin, and topiramate in the neonatal intensive care unit.

EEG, electroencephalography; HIE, hypoxic-ischemic encephalopathy; IVH, intraventricular hemorrhage; LEV, levetiracetam; MRI, magnetic resonance imaging; PB, phenobarbital; SWI, susceptibility-weighted imaging; USG, ultrasonography.

When neonates with no HIE were compared with those with any HIE, bicarbonate levels were significantly lower in the HIE group [8.4 (7.8–9.0) vs. 13.7 (11.1–14.8) mmol/L, *p* = 0.010]. The HIE group also tended to have lower blood gas pH and lower 5 min Apgar scores, although these differences did not reach statistical significance. No significant between-group differences were observed in sex, mode of delivery, gestational age, birth weight, seizure frequency, EEG abnormalities, neuroimaging abnormalities, or antiepileptic treatment use (see Table 4).

**Table 4:** Comparison of neonates with no HIE and any HIE.

Variable	No HIE (*n* = 16)	Any HIE* (*n* = 6)	*p* value
Male sex, *n* (%)	8 (50.0)	4 (66.7)	0.646
Cesarean delivery, *n* (%)	9 (56.2)	3 (50.0)	1.000
Gestational age, weeks	39.0 (38.0–40.0)	40.0 (39.3–40.0)	0.466
Birth weight, g	3,300 (3,040–3,465)	3,335 (2,985–4,210)	0.533
Apgar score at 1 min	4.0 (3.0–5.0)	3.5 (2.8–4.5)	0.914
Apgar score at 5 min	6.0 (5.3–7.5)	5.0 (3.8–6.3)	0.328
Blood gas pH	7.00 (6.90–7.10)	6.86 (6.80–6.90)	0.207
pCO_2_, mmHg	60.9 (60.0–62.9)	60.3 (41.4–85.9)	0.915
HCO_3_, mmol/L	13.7 (11.1–14.8)	8.4 (7.8–9.0)	**0** **.** **010**
Base excess	−14.1 (−17.2 to −12.7)	−17.6 (−20.7 to −13.2)	0.755
Seizures, *n* (%)	8 (50.0)	3 (50.0)	1.000
Abnormal EEG, *n* (%)	4 (25.0)	1 (16.7)	1.000
Abnormal cranial USG, *n* (%)	1 (7.1)	1 (33.3)	0.331
Abnormal cranial MRI, *n* (%)	11 (73.3)	3 (50.0)	0.354
Any antiepileptic treatment, *n* (%)	9 (56.2)	4 (66.7)	1.000

* Any HIE includes stage 1, stage 2, and stage 3 HIE.

Continuous variables are presented as median (IQR) and categorical variables as *n* (%). *P* values were calculated using the Mann–Whitney *U* test for continuous variables and Fisher's exact test for categorical variables. Because of missing data, denominators may vary across variables.

Bold values indicate statistically significant p-values (*p* < 0.05).

Compared with neonates without seizures, those with seizures had a lower gestational age [38.0 (38.0–39.0) vs. 40.0 (40.0–40.0) weeks, *p* = 0.002] and a more negative base excess [−20.3 (−24.5 to −16.2) vs. −13.0 (−14.9 to −9.5), *p* = 0.029]. Five-minute Apgar scores also tended to be lower in the seizure group, although this difference did not reach statistical significance. Antiepileptic treatment was significantly more frequent in neonates with seizures (100.0% vs. 18.2%, *p* < 0.001). No significant differences were found in sex, birth weight, HIE status, EEG abnormality, cranial USG abnormality, or cranial MRI abnormality (see Table 5).

**Table 5:** Comparison of neonates with and without seizures.

Variable	No seizure (*n* = 11)	Seizure (*n* = 11)	*p* value
Male sex, *n* (%)	5 (45.5)	7 (63.6)	0.670
Cesarean delivery, *n* (%)	4 (36.4)	8 (72.7)	0.198
Any HIE, *n* (%)	3 (27.3)	3 (27.3)	1.000
Gestational age, weeks	40.0 (40.0–40.0)	38.0 (38.0–39.0)	**0** **.** **002**
Birth weight, g	3,300 (3,120–3,500)	3,290 (2,600–3,482.5)	0.725
Apgar score at 1 min	4.0 (3.0–5.5)	2.5 (1.5–3.5)	0.251
Apgar score at 5 min	6.0 (5.5–7.5)	4.0 (2.8–5.3)	0.085
Blood gas pH	6.90 (6.81–7.03)	6.90 (6.85–7.00)	0.953
pCO_2_, mmHg	69.7 (60.5–102.6)	39.7 (35.1–48.1)	0.069
HCO_3_, mmol/L	11.0 (8.5–13.9)	9.2 (9.0–11.2)	0.876
Base excess	−13.0 (−14.9 to −9.5)	−20.3 (−24.5 to −16.2)	**0** **.** **029**
Abnormal EEG, *n* (%)	2 (18.2)	3 (27.3)	1.000
Abnormal cranial USG, *n* (%)	0 (0.0)	2 (18.2)	0.476
Abnormal cranial MRI, *n* (%)	7/10 (70.0)	7/11 (63.6)	1.000
Any antiepileptic treatment, *n* (%)	2 (18.2)	11 (100.0)	**<0** **.** **001**

Continuous variables are presented as median (IQR) and categorical variables as *n* (%). *P* values were calculated using the Mann–Whitney *U* test for continuous variables and Fisher's exact test for categorical variables. Because of missing data, denominators may vary across variables.

Bold values indicate statistically significant p-values (*p* < 0.05).

Concordance analysis between cranial ultrasonography and cranial MRI was performed in 13 neonates with both imaging modalities available (Table 6). Among these, cranial USG was normal in 11 patients, although 8 of them had abnormal MRI findings. Both cranial USG and MRI were abnormal in 2 patients, whereas no patient had abnormal cranial USG with a normal MRI. Overall, agreement between the two imaging modalities was low, suggesting that cranial MRI detected abnormalities not identified on cranial ultrasonography. Overall agreement was 38.5%, with a low Cohen's kappa coefficient (*κ* = 0.10).

**Table 6:** Concordance between cranial ultrasonography and cranial MRI findings.

Cranial USG findings	Normal MRI, *n*	Abnormal MRI, *n*	Total, *n*
Normal USG	3	8	11
Abnormal USG	0	2	2
Total	3	10	13

Only patients with both cranial USG and cranial MRI data available were included in the concordance analysis.

In exploratory univariable Firth penalized logistic regression analyses, higher bicarbonate levels were associated with lower odds of any-stage HIE. Each 1 mmol/L increase in bicarbonate was associated with an approximately 46% reduction in the odds of HIE (OR = 0.54, 95% CI: 0.27–0.86; *p* = 0.018). Blood gas pH, base excess, 5 min Apgar score, and gestational age were not significantly associated with any-stage HIE (Table 7).

**Table 7:** Firth penalized logistic regression analysis of factors associated With Any-stage hypoxic-ischemic encephalopathy.

Predictor	OR	95% CI	*p* value
HCO_3_, per 1 mmol/L increase	0.54	0.27–0.86	0.018
Blood gas pH, per 0.1-unit increase	0.63	0.31–1.12	0.118
Base excess, per 1 mmol/L increase	0.92	0.78–1.06	0.244
5 min Apgar score, per 1-point increase	0.73	0.42–1.18	0.203
Gestational age, per 1-week increase	1.21	0.63–2.61	0.578

OR, odds ratio; CI, confidence interval; HCO_3_, bicarbonate; HIE, hypoxic-ischemic encephalopathy.

Each predictor was evaluated in a separate univariable Firth penalized logistic regression model. ORs for continuous variables represent the change in the odds of any-stage HIE for each unit increase specified in the table. A two-sided *p* value of <0.05 was considered statistically significant.

In exploratory univariable Firth penalized logistic regression analyses, lower gestational age and more negative base excess values were significantly associated with seizure occurrence. Each additional week of gestation was associated with a 76% reduction in the odds of seizures (OR = 0.24, 95% CI: 0.07–0.59; *p* = 0.004). Each 1 mmol/L increase in base excess, reflecting less severe metabolic acidosis, was associated with a 16% reduction in seizure odds (OR = 0.84, 95% CI: 0.70–0.96; *p* = 0.017). The 5 min Apgar score and pCO_2_ showed borderline inverse associations with seizure occurrence but did not reach statistical significance. Bicarbonate level and the presence of any-stage HIE were also not significantly associated with seizures (Table 8).

**Table 8:** Firth penalized logistic regression analysis of factors associated With neonatal seizures.

Predictor	OR	95% CI	*p* value
Gestational age, per 1-week increase	0.24	0.07–0.59	0.004
Base excess, per 1 mmol/L increase	0.84	0.70–0.96	0.017
5 min Apgar score, per 1-point increase	0.72	0.49–1.01	0.064
pCO_2_, per 10-mmHg increase	0.71	0.47–1.02	0.072
HCO_3_, per 1 mmol/L increase	0.94	0.73–1.19	0.593
Any-stage HIE	1.00	0.20–4.91	1.000

OR, odds ratio; CI, confidence interval; HCO_3_, bicarbonate; HIE, hypoxic-ischemic encephalopathy; pCO_2_, partial pressure of carbon dioxide.

Each predictor was evaluated in a separate univariable Firth penalized logistic regression model. ORs for continuous variables represent the change in the odds of neonatal seizure occurrence for each unit increase specified in the table. A two-sided *p* value of <0.05 was considered statistically significant.

## Discussion

In this retrospective single-center cohort of neonates who underwent therapeutic hypothermia for suspected hypoxic-ischemic encephalopathy, the principal biochemical and clinical findings were twofold. First, bicarbonate levels were significantly lower in neonates with any stage of HIE than in those with no final evidence of HIE. Second, neonates with seizures had lower gestational age and a more negative base excess than those without seizures. As a secondary descriptive finding, cranial ultrasonography showed poor concordance with magnetic resonance imaging, with several infants having normal ultrasonography despite abnormal MRI findings. The association between lower bicarbonate levels and HIE is consistent with previous evidence linking metabolic acidosis to hypoxic-ischemic injury and should not be interpreted as a novel finding. Moreover, because the analysis compared infants with no final evidence of HIE with those with any-stage HIE, it does not establish an association between bicarbonate levels and increasing HIE severity. Taken together, the findings provide supportive real-world evidence that early acid-base disturbances may help characterize neurologic risk among neonates selected for therapeutic hypothermia. The observed USG–MRI discordance was consistent with the established greater sensitivity of MRI and was not considered a novel finding of the present study (8–11).

Our finding that bicarbonate levels were significantly lower in the HIE group is clinically plausible and consistent with the established role of metabolic acidosis as an early marker of perinatal hypoxic-ischemic insult. This association is not novel and should be interpreted as a supportive finding within a treatment-selected cohort of neonates who underwent therapeutic hypothermia. Moreover, because our analysis compared neonates with no final evidence of HIE with those with any-stage HIE, it does not demonstrate a graded relationship between bicarbonate levels and HIE severity. Previous literature has shown that severe acidosis, including low pH and marked base deficit, is associated with more severe encephalopathy and worse short-term and long-term outcomes. Therapeutic hypothermia is now the standard neuroprotective treatment for term and near-term infants with moderate to severe hypoxic-ischemic encephalopathy, but the decision to initiate cooling is still heavily influenced by early biochemical and clinical indicators of perinatal compromise. In this context, the lower bicarbonate levels observed in infants with any HIE in our cohort provide confirmatory, real-world evidence that early acid-base disturbance may remain a useful adjunct marker for clinical risk characterization among cooled neonates (8, 10, 11).

Although blood gas pH and 5 min Apgar scores tended to be lower in neonates with hypoxic-ischemic encephalopathy, these differences did not reach statistical significance. This may reflect the small sample size and the heterogeneity of a cohort selected on the basis of receiving therapeutic hypothermia rather than final HIE classification alone. Importantly, neonatal encephalopathy and HIE are related but not identical entities, and not all cooled infants ultimately demonstrate sufficient evidence to support a final diagnosis of hypoxic-ischemic brain injury. This is particularly relevant to the present study, in which many infants who underwent therapeutic hypothermia were subsequently classified as having no final evidence of HIE. Cooling had been initiated within the narrow therapeutic window on the basis of suspected hypoxic-ischemic injury, whereas the final classification was determined after consideration of the subsequent clinical course, serial neurologic assessments, electroencephalographic findings, and neuroimaging results (10, 12, 13). Importantly, the no-final-evidence-of-HIE group should not be interpreted as a healthy or untreated control group. All infants had initially been considered at sufficient risk of hypoxic-ischemic brain injury to justify therapeutic hypothermia. Therefore, the comparison reflects differences within a treatment-selected cohort rather than the diagnostic performance of bicarbonate or other blood gas parameters in the general neonatal population.

Another notable finding was the association of seizures with lower gestational age and more negative base excess. The association between more severe metabolic disturbance and seizure occurrence is biologically reasonable, as seizures in neonatal encephalopathy are generally considered a manifestation of more severe or more active brain injury. Recent studies in cooled infants with HIE have shown that seizures remain common despite therapeutic hypothermia, although seizure prevalence may be lower than in historical pre-cooling cohorts. In our cohort, antiepileptic therapy was, unsurprisingly, much more frequent in the seizure group. However, electroencephalographic abnormalities were not significantly different between groups, likely because electroencephalographic data were incomplete and not uniformly available in all patients. This limited sensitivity of bedside electroencephalographic categorization should be interpreted cautiously, particularly because prior studies have demonstrated that structured electroencephalographic assessment may provide prognostic information even after controlling for MRI findings (14, 15).

The neuroimaging findings provide additional descriptive information regarding the heterogeneity of this cohort. In the no HIE group, cranial MRI still demonstrated heterogeneous abnormalities, including hemorrhagic, ischemic, white matter, basal ganglia, and posterior fossa lesions. This again underscores the complexity of cooled neonatal encephalopathy and the fact that the final bedside HIE stage may not fully capture all structural abnormalities. In contrast, both infants with stage 3 HIE had marked MRI abnormalities and seizures, which is in line with the established relationship between more severe encephalopathy and greater structural brain injury. Prior studies and reviews have consistently shown that MRI is one of the most valuable tools for defining the pattern and severity of injury after neonatal encephalopathy and for estimating prognosis in infants treated with hypothermia (8, 9, 16).

A secondary finding of the present study was the low agreement between cranial ultrasonography and magnetic resonance imaging. Among infants with both imaging modalities available, cranial ultrasonography was normal in many cases in which MRI was abnormal, resulting in low overall agreement and a very low Cohen's kappa coefficient. This observation does not establish a novel superiority of MRI but is consistent with previous evidence regarding the limitations of cranial ultrasonography in detecting the full spectrum of neonatal brain abnormalities. Sanislow et al. reported that many abnormalities seen on early cranial ultrasonography did not correspond to later MRI findings, while intracranial hemorrhages identified on MRI were frequently missed by ultrasonography (17). More broadly, current reviews of neonatal encephalopathy imaging emphasize that ultrasonography has important practical advantages but remains less sensitive than MRI for defining the full extent and pattern of hypoxic-ischemic injury, especially when injury involves deep gray nuclei, white matter, or more subtle diffusion abnormalities (8, 9). Accordingly, the USG–MRI comparison in our cohort should be interpreted as a supportive, real-world observation rather than as a novel contribution.

Beyond conventional blood gas parameters, near-infrared spectroscopy (NIRS)-derived regional cerebral oxygen saturation (CrSO_2_) has emerged as a promising noninvasive bedside marker of cerebral oxygenation in neonates with perinatal asphyxia. Kazanasmaz et al. compared 42 neonates with moderate-to-severe encephalopathy undergoing therapeutic hypothermia with 42 healthy term neonates and demonstrated significantly lower bilateral CrSO_2_ values in the asphyxia group. They also reported moderate positive correlations between cord blood gas pH and both right and left cerebral oxygen saturation measurements, suggesting that systemic acid–base derangement and cerebral tissue oxygenation provide related but complementary information. A left-sided CrSO_2_ cutoff of ≤72% showed potentially useful diagnostic performance in differentiating affected neonates from healthy controls. These findings indicate that integrating NIRS-derived cerebral oxygenation measurements with cord or early postnatal blood gas parameters may improve early identification of clinically relevant cerebral hypoxia and provide a more direct assessment of cerebral oxygen balance during therapeutic hypothermia. NIRS measurements were not available in our retrospective cohort; therefore, we could not determine whether reduced bicarbonate levels or more negative base excess values were accompanied by impaired regional cerebral oxygenation. Future prospective studies combining serial blood gas analysis, continuous NIRS monitoring, electroencephalography, and MRI are warranted to clarify the incremental diagnostic and prognostic value of multimodal neuromonitoring in cooled neonates (18).

This study has several limitations. First, it was retrospective and conducted at a single center with a limited sample size, which restricts statistical power and generalizability. Second, some laboratory, electroencephalographic, ultrasonographic, and MRI data were missing, and denominators therefore varied across analyses. Third, the cohort was defined by therapeutic hypothermia exposure rather than a uniform final diagnosis of definite hypoxic-ischemic encephalopathy, introducing clinical heterogeneity. Final HIE classification was assigned retrospectively after review of the complete clinical course and was not based exclusively on a prospectively standardized neurologic examination performed before the initiation of cooling. Therefore, some degree of diagnostic misclassification cannot be excluded. Moreover, the group classified as having no final evidence of HIE was not a healthy or untreated control group but consisted of neonates initially cooled because of suspected hypoxic-ischemic injury. Consequently, comparisons between the no-final-evidence-of-HIE and any-HIE groups should be regarded as exploratory comparisons within a treatment-selected cohort. Fourth, neurodevelopmental follow-up data were incomplete and could not be analyzed robustly. In addition, although blood gas sampling was performed within the first hour after birth and before the initiation of therapeutic hypothermia, and MRI was generally obtained after rewarming, the exact timing of these assessments was not identical in all neonates because of the retrospective nature of the study, which may have influenced the observed associations. The blood gas analysis included both umbilical cord arterial samples and early postnatal arterial samples. Although no venous or capillary samples were included, differences in sampling time and physiologic conditions between cord arterial and postnatal arterial measurements may have introduced some measurement heterogeneity. Furthermore, regional cerebral oxygen saturation was not monitored using NIRS; consequently, the relationship between systemic acid–base abnormalities and direct bedside measures of cerebral oxygenation could not be evaluated. Nevertheless, the study also has several strengths, including detailed extraction of biochemical, clinical, electroencephalographic, treatment, and neuroimaging variables from a real-world cohort of cooled neonates and the integrated evaluation of early blood gas parameters, final HIE classification, and seizure occurrence.

## Conclusion

In conclusion, our findings suggest that lower bicarbonate levels may be associated with the presence of any-stage HIE compared with no final evidence of HIE in neonates undergoing therapeutic hypothermia for suspected perinatal hypoxic-ischemic injury, while more negative base excess may be associated with seizure occurrence. The bicarbonate finding is consistent with previously established evidence regarding metabolic acidosis in hypoxic-ischemic injury and should be interpreted as confirmatory and supportive rather than novel. Because the analysis did not evaluate a graded relationship across HIE stages, no conclusions can be drawn regarding bicarbonate levels and increasing HIE severity. The low concordance observed between cranial ultrasonography and magnetic resonance imaging was consistent with previous evidence regarding the greater sensitivity of MRI and should be interpreted as a supportive secondary finding rather than a novel contribution of the present study. Given the small, treatment-selected cohort and retrospective final classification, these associations should be regarded as exploratory. Larger prospective studies with standardized electroencephalographic monitoring and longitudinal neurodevelopmental follow-up are needed to clarify the prognostic value of early blood gas parameters and multimodal neurologic assessment in cooled neonates with suspected or confirmed hypoxic-ischemic encephalopathy.

## Data Availability

The raw data supporting the conclusions of this article will be made available by the authors, without undue reservation.
